# Reference Values and Effect of Age on Hemogram in Landim Cattle Raised in Extensive System in Districts of Xai-Xai, Limpopo, and Chongoene, Gaza Province, Mozambique

**DOI:** 10.3390/vetsci12121124

**Published:** 2025-11-27

**Authors:** Carlos Francisco Macuvele, Atanásio Serafim Vidane, Daniela Becker Birgel, Ana Claudia Oliveira Carreira Nishiyama, Eduardo Harry Birgel Junior

**Affiliations:** 1 Faculty of Veterinary Medicine and Animal Science, Save University, Chongoene 1200, Mozambique; 2 Department of Clinic and Surgery, Faculty of Veterinary Medicine and Animal Science, University of São Paulo, São Paulo 05508-010, Brazil; 3 Department of Clinical Medicine, Faculty of Veterinary Medicine, Eduardo Mondlane University, Maputo 1109, Mozambique; atanasiovidane@gmail.com; 4 Department of Veterinary Medicine, Faculty of Animal Science and Food Engineering, University of São Paulo, Pirassununga 13635-900, Brazil; dabirgel@usp.br; 5 Center for Natural and Human Sciences (CCNH), Federal University of ABC, São Bernardo do Campo 09606-045, Brazil; anaclaudiaoli@gmail.com

**Keywords:** age factor, erythrogram, leukogram, cattle, Nguni

## Abstract

This research aimed to establish reference intervals and evaluate the influence of age in 56 Nguni cattle raised in Mozambique. Blood samples containing EDTA as anticoagulant were collected. The erythrogram and total leukocyte count were analyzed using the BC-2800 Vet Mindray^®^ automatic counter. The differential counting of leukocytes was performed in blood smears stained using Giemsa and Mcgruwald’s stain. Statistical analysis was performed using the statistical analysis system (SAS). Analysis of variance was performed using the GLM procedure, and mean contrasts were analyzed using Duncan’s parametric test at 5% significance, with the Shapiro–Wilk and Levene tests for date normalities. The reference intervals for the erythrogram are as follows: red blood cells: 6.78 to 7.40 × 10^12^/L; hemoglobin: 10.77 to 11.36 g/dL; hematocrit: 28.02 to 29.56%; mean corpuscular volume (MCV): 39.91 to 43.02 fL; mean corpuscular hemoglobin (MCH): 15.27 to 16.44 pg; mean corpuscular hemoglobin concentration (MCHC): between 37.86 and 39.10 g/dL; and red blood cell distribution width (RDW): between 16.98 and 19.40%. For leukograms, the following references values were obtained: total leukocytes: between 14,106 and 16,233 × 10^6^/L; basophils: between 32 and 165 × 10^6^/L; eosinophils: between 823 and 1262 × 10^6^/L; band neutrophils: between 25 and 87 × 10^6^/L; segmented neutrophils: between 2510 and 3249 × 10^6^/L; total neutrophils: between 2565 and 3306 × 10^6^/L; lymphocytes: between 9471 and 11,474 × 10^6^/L; and monocytes: between 154 and 296 × 10^6^/L. Age influenced MCV, MCH, leukocytes, eosinophils, and lymphocytes. Leukogram reference intervals of other countries could not be used for the breed of Mozambique without making gross errors.

## Simple Summary

Hemogram values obtained for animals reared in a given region cannot be considered, without proper evaluation, as a reference standard for other locations. Reference values have not been established for the hemogram of the Landim breed. Thus, the objective of this study was to establish reference values of the hemogram of the Landim breed and evaluate the effect of age in cattle raised in Mozambique in the extensive sector. Fifty-six animals were included in this study, subdivided by age. For these animals, reference intervals were established, and with the evaluation of the effect of age, it was found that age influenced the mean corpuscular volume, mean corpuscular hemoglobin, leukocytes, lymphocytes, and eosinophils. This study may help veterinarians in clinical diagnosis based on the changes observed in an animal’s blood cells and, thus, to take a more assertive and timely attitude in disease control, thus reducing cattle mortality in Mozambique.

## 1. Introduction

Veterinary hematology is an auxiliary means for veterinarians to diagnose, monitor, and determine appropriate treatment for numerous diseases and to establish prognoses regarding the future evolution of a disease in an individual [1,2]. Animals raised in different environmental, climatic, and management conditions present evident variations in blood elements, and the values obtained for animals raised in a given region cannot be considered, without adequate evaluation, as a reference standard for other locations [2,3,4,5].

In regions where Anaplasma and Babesia species are endemic and where enzootic stability is present, animals often exhibit leukocytosis, primarily due to lymphocytosis [3]. As a result, the standard blood count reference values established in the Northern Hemisphere are not suitable for use in these tropical and subtropical areas of the Southern Hemisphere. It has been recommended that laboratories should establish their own reference ranges that reflect current cattle populations [1]. Applying these reference values can lead to serious errors in interpreting blood tests, thereby invalidating their use in these contexts [3,4].

On the African continent, theileriosis should be recognized as a significant tick-borne disease [6,7,8]. In Mozambique, the primary causative agent is *Theileria parva*, which infects both lymphocytes and red blood cells. This infection leads to a clinical presentation characterized by fever, anemia, jaundice, hemoglobinuria, and enlargement of the superficial lymphnodes [9,10,11]. The phenomenon of enzootic stability for *Theileria* spp. has been documented across various regions in Africa [6,7,8]. It is therefore reasonable to assume that enzootic stability for theileriosis may also influence the reference hematological values in cattle raised under such enzootic conditions.

The Landim (Nguni) breed, native to Central and East Africa, is the predominant breed in the family farming sector in Mozambique [12]. Although it belongs to the *Bos taurus* species, its morphological characteristics led it to be considered a different subspecies from the taurines (*Bos taurus taurus*) and zebu cattle (*Bos taurus indicus*), with its scientific classification being *Bos taurus africanus* [13,14]. This breed was recognized as important due to its robustness and disease resistance in 1988 [12].

A review of the literature revealed no studies on reference intervals or the effect of age on the blood count of native cattle raised on the African continent, specifically in Mozambique, such as the Landim (Nguni) breed of subspecies *Bos taurus africanus* used in this study. Therefore, this study aims to establish reference values and evaluate the effect of age on the blood count of Nguni-Landim (*Bos taurus africanus*) cattle in the districts of Xai-Xai, Limpopo, and Chongoene, located in Gaza Province, Mozambique.

## 2. Materials and Methods

The Animal Use Ethics Committee of the School of Veterinary Medicine and Animal Science of the University of São Paulo (protocol number 1548080523) approved all the animal procedures. This research was conducted in partnership between the University of São Paulo (USP) in Brazil and Save University in Mozambique.

This study was conducted on small rural properties with extensive livestock breeders in Mozambique. Blood samples were collected from 140 animals, of which only 56 were considered healthy animals, to establish reference blood count values.

All animals were examined, and sick animals or those with clinical signs observed in theileriosis, such as fever, anemia, jaundice, hemoglobinuria, or lymphadenomegaly, were excluded [9,10,11]. When evaluating the erythrogram, anemic animals were excluded. Only cows that had red blood cell, hematocrit, and hemoglobin values of ≥ 5.0 × 10^12^/L, 24%, and 8.0 g/dL were classified as non-anemic and were used [15]. In all animals used, the blood smears did not show intra-erythrocytic parasites, and the absence of jaundice ruled out the possibility that the animals’ disease was caused by hemoparasites [16].

### 2.1. Description of the Study Site

**Figure 1:**
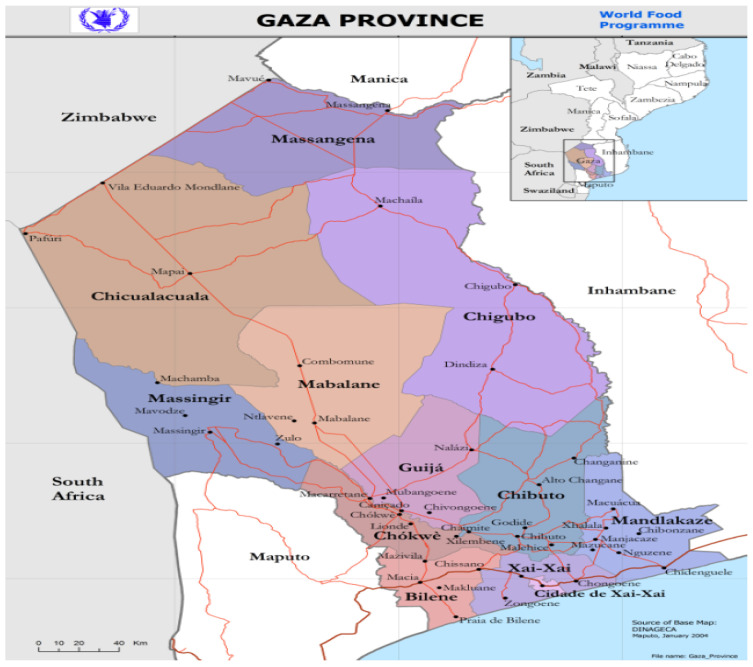
Map of Gaza province, indicating the districts of Chongoene, Xai-Xai, and Limpopo. Source: [17].

The animals used were from the 3 districts of Xai-Xai, Chongoene, and Limpopo (see Figure 1) of the Gaza province in Mozambique, kept in an extensive or family farming system. The three districts are located in the southern part of Gaza Province. Xai-Xai District is geographically bounded by Chibuto District to the north, Manjacaze to the east, the Indian Ocean to the south, and Bilene Macia to the west. Its geographic coordinates are as follows: latitude: 25,003′06″ S; longitude: 33,038′39″ E; altitude above sea level: 4 m. Limpopo District is geographically bounded by Chibuto District to the north, Chongoene and Xai-Xai to the east, the Indian Ocean to the south, and Bilene to the west. Its geographic coordinates are as follows: latitude: 210 and 250″ S; longitude: 310 and 350″. Chongoene District is geographically bounded by Chibuto District to the north, Manjacaze to the east, the Indian Ocean to the south, and Limpopo to the west. Its geographic coordinates are as follows: latitude: 25,001′10″ S; longitude: 33,047′46″ E [17]. The average annual temperature is around 25.5 °C, with typical highs of 31.6 °C and lows of 20.2 °C. The region receives approximately 1183 mm of rainfall per year, most of which occurs during the summer months. The average relative humidity is about 73 percent.

### 2.2. Sample Population and Procedures

The predominant cattle farming system in Mozambique is extensive, with feed management based on natural pastures of *Panicum maximum*, *Cynodon dactylon*, *Setaria sphacelata*, *Chloris gayana*, *Themeda triandra*, or *Heteropogon contortus*. All animals exclusively feed on the natural pasture used.

Fifty-six female healthy animals of Landim breed were used and divided into six groups according to age and the stage of their productive life: G1—seven suckling calves up to 6 months old; G2—seven weaned calves aged between 6 and 12 months; G3—eleven pubertal heifers aged between 12 and 24 months; G4—ten primiparous cows aged between 24 and 48 months; G5—thirteen animals aged between 48 and 72 months with two calvings; G6—eight animals over 72 months with three or more calvings. These groups were used to establish references ranges and evaluate the influence of age on the hematological parameters.

### 2.3. Laboratory Analysis

Blood samples were collected from cattle before they were released into the natural pasture, between 6 and 8 a.m., via puncture of the coccygeal vein using the Vacutainer^®^ System, Becton Dickinson, Franklin Lakes, NJ, USA. For hematological analysis, samples were collected in tubes containing ethylenedia-minetetraacetic acid (EDTA). The erythrogram (number of red blood cells, packed cell volume, hemoglobin content, mean corpuscular volume (MCV), mean corpuscular hemoglobin (MCH), mean corpuscular hemoglobin concentration (MCHC) and total leukocyte count was analyzed using the automatic hematology counter (Mindray^®^ BC-2800 Vet, Shenzhen, Guangdong, China) at the hematology laboratory of the School of Veterinary Medicine of Eduardo Mondlane University. With whole blood, blood smears were made for the differential leukocyte count. The slides with the blood smears were left to dry naturally and then stained with Giemsa and McGruwald staining.

In each blood smear, 100 leukocytes were differentiated, classified according to their morphological and staining characteristics as neutrophils with band nuclei, neutrophils with segmented nuclei, eosinophils, basophils, lymphocytes, and monocytes.

### 2.4. Statistical Analysis

Statistical analysis was performed using the statistical analysis system, SAS^®^, version 9.3 SAS/STAT; SAS Institute Inc., Cary, NC, USA. Residual normality tests were performed using the Shapiro–Wilk test, and the Levene test was used to assess the homogeneity of variance at a probability level of 5%.

Data with a non-normal distribution had their outliers removed (≥mean ± 2 standard deviation). This was carried out independently for each parameter instead of removing all data of an individual cow after detecting abnormal data. Supplementary Materials Figure S1 present the histograms and box plots of all variables. All variables, with the exception of band neutrophils and basophils, followed a Gaussian distribution (assumptions of the normality of the residuals and homogeneity of variance were accepted).

Analysis of variance was performed using the GLM procedure to test the equality of means, and mean contrasts were analyzed using the Duncan test with significance levels equal to 5% (*p* < 0.05) [18]. To establish references range values, the 95% confidence interval for each variable was calculated using ASVCP reference interval guidelines [19].

## 3. Results

After statistical analysis, the reference intervals of the erythrogram and leukogram in Nguni or Landim cattle raised in Mozambique were obtained (Table 1) in relation to their mean, standard error of mean (SEM) and 95% confidence interval. Based on the confidence interval, the reference values for the erythrogram and leukogram are presented in Table 1.

### Influence of Age on Hemogram of Landim Breed

As can be seen in Table 2, the values of the number of red blood cells (*p* values = 0.2652), hemoglobin rate (*p* values = 0.3700), and hematocrit (*p* values = 0.3712) were not influenced by age factors and ranged between 6.49 ± 0.25 and 7.54 ± 0.47 × 10^12^/L red blood cells/µL, 10.45 ± 0.46 and 11.65 ± 0.19 g/dL, and 27.50 ± 0.75 and 30.26 ± 1.06%, respectively.

Regarding the absolute hematimetric indices (Table 2), it was observed that MCV was influenced by age (*p* values = 0.048). The values observed in the group of calves up to six months old (37.20 ± 0.94 fL) increased with age, reaching the highest values in the group of animals aged 48 to 72 months (43.63 ± 1.04 fL), and those over 72 months of age (47.06 ± 2.16 fL) were significantly higher. The mean corpuscular hemoglobin values (*p* values = 0.0006) were influenced by age and exhibited a gradual increase, which went from 14.00 ± 0.50 pg (observed in the group of animals up to six months old) to 14.85 ± 0.68 pg (observed in animals aged between 24 and 48 months) and 18.05 ± 0.63 pg (in animals over 72 months of age). MCHC was not influenced by age (*p* values = 0.4673) and ranged from 37.62 ± 0.95 to 39.52 ± 0.91 g/dL. Likewise, no influence of age was observed on RDW values, which ranged between 16.73 ± 0.50 and 19.43 ± 1.91%, without evident statistical differences (*p* values = 0.7294).

Regarding the leukogram (Table 3), the total number of leukocytes increased from 12,557 ± 1734 × 10^6^/L, observed in the group of calves up to six months old, to 18,214 ± 1646 × 10^6^/L, observed in the group with ages ranging from 6 to 12 months of age. Then, a gradual decrease in the total number of leukocytes was observed, reaching minimum values equal to 13,912 ± 1278 ×10^6^/L in the group of animals over 72 months old (*p* value = 0.0232).

The absolute number of lymphocytes was observed to increase from 9493 ± 1620 × 10^6^/L, in the group of calves up to six months old, to 13,252 ± 1781 × 10^6^/L in the group ranging in age from 6 to 12 months. Then, a gradual decrease in the lymphocyte number was observed, reaching minimum values equal to 8074 ± 690 × 10^6^/L in the group of animals over 72 months old (*p* value = 0.0283).

As can be seen in Table 3, the total number of neutrophils (*p* value = 0.2127) was not influenced by age and ranged, respectively, between 2336 ± 356 and 4008 ± 765 neutrophils × 10^6^/L. Likewise, an influence of age on the total number of band (*p* value = 0.4936) and segmented neutrophils (*p* values = 0.1700) was not observed (Table 3).

The absolute numbers of eosinophils (Table 3) observed in the group of animals aged 48 to 72 months (1216 ± 198 eosinophils × 10^6^/L) and over 72 months (1588 ± 199 eosinophils × 10^6^/L) were significantly higher (*p* values = 0.0470) than those observed in the group under 6 months (359 ± 154 eosinophils × 10^6^/L). As can be seen in Table 3, the absolute numbers of basophils (*p* values = 0.2088) and monocytes (*p* values = 0.1975) were not influenced by age factor and ranged, respectively, between 0 and 280 ± 191 basophils × 10^6^/L and between 87 ± 59 and 310 ± 77 monocytes × 10^6^/L, without statistical differences between the groups observed.

## 4. Discussion

The results obtained in this study were closer to those reported for zebu breeds (*Bos taurus indicus*) raised in the Southern Hemisphere [20,21,22]. The results of the reference values of the hemogram for Nguni (Landim) cattle raised in Mozambique differ from those reported for the taurine (*Bos taurus taurus*) breed raised in the Northern Hemisphere [23,24,25]. Table 4 compares the results of the Jersey cattle [3,4], Holstein cattle [23,24,25], and Nelore zebu cattle [20,21]. Few studies [23,24,25] have established reference values for the blood count according to the American Society for Veterinary Clinical Pathology (ASVCP), which recommends adherence to the guidelines of the Clinical Laboratory and Laboratory Standards Institute (CLSI) for the determination of population-based reference intervals [19].

CSLI recommends nonparametric statistical analysis, because the majority of clinical chemical analytes do not follow a normal distribution; however, when sampling varies between 40 and 120, the reference interval can be established using a parametric test if a Gaussian distribution can be established [19]. It was shown that 13 of 18 analytes for the blood count in the reference population were normally distributed, and 2 additional analytes were readily converted to a normal distribution via square root transformation [24]. The CLSI indicates that parametric methods for smaller sample sizes can be used to establish the reference interval [19]. Our sample size of 56 is larger than the recommended minimum for normally distributed populations (40). The distances between the farms used and the Save University laboratory justify our sample size. For a veterinary laboratory located far from numerous beef or dairy farms, such a large number of good-quality samples would be difficult to obtain and expensive to analyze [24].

Given the importance of theileriosis as a bovine disease in Mozambique, causing fever, anemia, jaundice, and hemoglobinuria [9,10,11], it was expected that there would be more significant changes in the erythrogram, with the reference values being significantly different from those adopted outside the African continent for several cattle breeds [3,4,20,21,23,24,25]. Similar to what was reported for endemic areas of *Anaplasma* sp. and *Babesia* spp., where there is enzootic stability [3,4], the reference values for the erythrogram used in the Northern Hemisphere can be used without making gross errors for Landim cattle. It is possible that the districts of Xai-Xai, Chongoene, and Limpopo in which we collected the blood samples are in areas of enzootic stability of *Theileria parva*. In such areas, a reduction in the number of clinical cases of theileriosis has been considered an indicator of the occurrence of enzootic stability [9,26].

Although there are no epidemiological studies on theileriosis in Mozambique, it is likely that there is enzootic stability, as areas of enzootic stability for Theileria have been observed on the African continent [6,7,8]. Enzootic stability is a widely used term in the epidemiology of ticks and tick-borne diseases [27]. The main features of enzootic stability in this context are a high level of challenge with hemoparasite-infected ticks and a concurrent low incidence of clinical disease [27]. It refers to a state where there is widespread parasite transmission and a high number of infected, asymptomatic animals, which leads to a low incidence of clinical disease in the population [27]. In regions with enzootic stability of hemoparasites such as Babesia, Anaplasma, and Theileria, it is estimated that more than 75% of the population circulate the parasite, meaning they are infected but not sick [28]. The established reference values assume that animals raised in Mozambique and throughout Africa are under enzootic stability for tick-borne diseases.

The exclusion of animals with clinical signs observed in theileriosis [9,10,11], the exclusion of anemic animals [15], and the absence of intra-erythrocytic parasites in blood smears [16] ensure that only healthy animals were used to establish reference values.

The total number of leukocytes and absolute number of lymphocytes found in Landim animals raised in Mozambique were higher (between 25% and 400% for leukocytes and 200 to 500% for lymphocytes) than those reported for taurine cattle raised in the Northern Hemisphere above a latitude of 32° [24,25]. The mean values for the total number of leukocytes (15,146 ± 531 leukocytes × 10^6^/L) and absolute number of lymphocytes (10,549 ± 500 lymphocytes × 10^6^/L) for the Landim breed are higher than the reference values in the Northern Hemisphere above a latitude of 32° recommended in the literature [24,25] and ranged between 4400 and 12,000 leukocytes × 10^6^/L and between 1600 and 6200 lymphocytes × 10^6^/L.

In subtropical regions, where cattle were infested by ticks and hemoparasitosis caused by *Anaplasma marginale* and *Babesia* spp. are enzootic diseases, a leukogram characterized by leukocytosis with lymphocytosis is observed [4]. In regions where hemoparasites are sporadic, such as in animals raised in the Northern Hemisphere (temperate or cold climate regions), it is observed that the total number of leukocytes and lymphocytes is lower [1,23,25]. Anaplasmosis and Babesiosis are diseases diagnosed in Mozambique [29], although the region is at the same latitude and has a similar climate to that in the study carried out in the southeast and central–west regions of Brazil [4,5,22]. It was observed that the numbers of leukocytes and lymphocytes were higher in the Landim breed raised in Mozambique than those reported for taurine [4,5] and zebu [22] breeds raised in the southeast and central–west regions of Brazil.

On the African continent, in addition to these hemoparasitoses (Anaplasmosis and Babesiosis), we have also observed theileriosis, which is responsible for a clinical picture that specifically affects the lymphatic system and lymphocytes [11,30,31,32]. This is expected to influence the number of lymphocytes and leukocytes. Changes in the leukogram are probably manifestations of mesological variations [3,4] and the influence of pathological pictures determined by *Theileria parva* [11,30,31,32] that do not exist in other subtropical regions. Unlike in other countries, the tick genera that infest cattle in Mozambique are diverse, such as Amblyomma, Dermacentor, Boophilus, Rhipicephalus, and Hyalomma, which are mentioned in several studies [30,31,32], these diversity may be associated with the particularities observed in the hemogram of Landim cattle.

When comparing the number of neutrophils obtained in this research with the values cited in the literature, it was found that there was equivalence with those presented for zebu breeds [21] raised in the Southern Hemisphere. In dairy breeds, such as Jersey [4] or Holstein [23,24,25], it was observed that the range of reference values (between 700 and 6800 neutrophils × 10^6^/L) was greater than that observed for the Landim breed (between 2510 and 3249 neutrophils × 10^6^/L).

The eosinophil values observed for Landim cattle were found to be higher than those reported for the taurine breed raised in the Southern Hemisphere [4] and Northern Hemisphere [24]. However, in some studies, the reference range for eosinophils was very large (between 100 and 2100 × 10^6^/L), and thus, the reference values for the Landim breed were within the range proposed for Nelore cows [21] raised in the Southern Hemisphere and Holstein cows [23,25] raised in the Northern Hemisphere. All Landim cattle used were healthy, with no clinical signs of helminth infections. However, preventive parasitic control is not performed on farms in Mozambique. The occurrence of eosinophilia, particularly in the group over 72 months old, could be explained as a type 1 hypersensitivity reaction or parasitic infections [1]. An increase in the number of circulating eosinophils in the blood has been correlated with low opg values in sheep feces, and this phenomenon has been related to greater animal resistance to parasitic infection [33]. In modern breeds in parasite control programs, with decreasing exposure to parasites, eosinophil counts are expected to be lower [1]. Eosinopenia has also been reported in situations where hemolytic anemia occurs and in acute infections by *Theileria* spp. [1]. The fact that we did not observe animals with eosinopenia confirms that there were no acute cases of theileriosis in the herds used, which is another indicator of enzootic stability for *Theileria parva* in cattle raised in Gaza province, in the Xai-Xai, Chongoene, and Limpopo Districts of Mozambique.

Comparing the number of basophils obtained in this research with the values cited in the literature reveals equivalence with those presented for taurine cattle raised in the Northern Hemisphere [23,24,25], as well as with those for taurine [4,5] and zebu breeds [21] raised in the Southern Hemisphere.

Comparing the number of monocytes obtained in this research with the reference values cited in the literature reveals equivalence with those presented for taurine cattle raised in the Southern Hemisphere [4], that they are higher than those for Zebu cattle raised in the Southern Hemisphere [21], and that they are lower than those for taurine cattle raised in the Northern Hemisphere [23,24,25].

Based on leukocyte and lymphocyte variations, it was considered correct to state that the leukogram reference values established in the Northern Hemisphere and other subtropical regions of the Southern Hemisphere should not be used to support clinical diagnosis in Mozambique.

Age, sex, stress, diet, body condition, reproductive status, recent activity, hydration, ambient temperature, and altitude are factors that contribute to physiological variations in the constituents of the blood count [1,2].

The evaluation of the effect of the age factor on the erythrogram and leukogram reveals that some variables were influenced, such as the MCV and MCH in the erythrogram and leukocytes, eosinophils, and lymphocytes, as described below. Variables such as hemoglobin, red blood cells, hematocrit, MCHC, and RDW were not influenced by age; that is, they did not present a statistically significant difference based on Duncan’s test at 5% probability.

The MCV of cattle in the study was influenced by the age factor between the animal groups, a result in agreement with that found by other authors who found a statistically significant difference, such as when studying the hematological profile of the Nelore breed [20], Jersey breed [3], Holstein [23] and Curraleiro breed [5]. The difference in VCM is caused by deficiencies in minerals such as iron or copper. Otherwise, regenerative anemia caused by difficulty in accessing natural pasture during periods of scarcity may also be associated with this [34]. Age influenced the mean corpuscular volume of bovine females of Aquitanian breed, and it was reported to increase significantly with the age of the animals [35]. A similar variation has been reported by other researchers in the same line of research [36]. Some researchers did not verify the influence of age on the mean corpuscular volume [13]. The mean corpuscular hemoglobin of Nguni or Landim cattle was influenced by the age factor, and some authors found similar results in the same line of research [35]. Other studies have shown, in turn, that age has influence on MCH. In other research, MCH exhibits a significant decrease in animals up to 3 to 11 months of age (12.67 ± 1.14 pg), and then an increase with an increasing age of cattle up to maximum values of (15.45 ± 1.01 pg) in animals over 60 months, such as the erythrogram of Holstein [5,36]. Another author reported in his research an increase in MCH in animals up to 24 and 48 months and that there is a constant and significant decline in values thereafter with increasing age [4]. The total leukocytes of Nguni or Landim cattle were influenced by age, similar to that observed for the effect of the age factor on the leukogram of female zebu cattle of the Nelore breed [22], in healthy Curraleiro cattle [5], Jersey breed [4], and Pantaneira breed [36]. In cattle, the total number of WBCs decreases with age [1]. It was proposed that the age-related decline in the total leukocyte count is a consequence of increased oxidative stress [37] (FINKEL, 2005). This oxidative damage leads to alterations in membrane composition and function, ultimately triggering cellular demise and a reduction in the number of leukocytes. With advancing age, the accumulation of free radicals compromises the integrity of cell membranes [37].

The lymphocytes of Nguni cattle were influenced by the age factor, in agreement with other researchers [4,5,36,38]. The increase in lymphocytes observed in their first 6 months of life can be justified through the increase in lymphocytes during the growth phase of the animals combined with the immunogenic activity in their first months of life and their decline in adulthood caused by the reduction in T lymphocytes due to the decrease in the thymus.

Eosinophils were influenced by age, as the averages showed a statistically significant difference. The number of these cells varied in all established groups. Researchers have also observed in their research that the age of the animal influences the eosinophil values [4,5,22,36]. This increase in eosinophils in adult animals is the result of memory immunity, particularly after parasitism [1,33].

## 5. Conclusions

Based on the observed variations in leukocytes and lymphocytes, it is appropriate to conclude that the leukogram reference values established for the Northern Hemisphere and other subtropical regions of the Southern Hemisphere should not be utilized to support clinical diagnosis in Mozambique. It is necessary to adopt local reference values obtained under African conditions.

The reference values for erythrograms adopted outside the African continent for various breeds of cattle can be used in areas endemic to theileriosis, where there is enzootic stability, without making gross errors in the interpretation of the erythrogram.

## Figures and Tables

**Table 1:** Reference intervals of erythrogram and leukogram of Nguni or Landim cattle, raised in Mozambique, according to statistical characteristics (mean, standard error of mean and 95% confidence interval).

Variable	Mean ± SEM	References Values (95% Confidence Interval)
Red blood cells (10^12^/L)	7.09 ± 0.15	6.78–7.40
Hemoglobin (g/dL)	11.07 ± 0.14	10.77–11.36
Hematocrit (%)	28.79 ± 0.37	28.02–29.56
MCV (fL)	41.47 ± 0.77	39.91–43.02
MCH (pg)	15.86 ± 0.29	15.27–16.44
MCHC (g/dL)	38.48 ± 0.31	37.86–39.10
RDW (%)	18.19 ± 0.60	16.98–19.40
Leukocytes (10^6^/L)	15,169 ± 531	14,106–16,233
Basophils (10^6^/L)	98 ± 33	32–165
Eosinophils (10^6^/L)	1042 ± 106	823–1262
Band neutrophils (10^6^/L)	56 ± 15	25–87
Segmented neutrophils (10^6^/L)	2879 ± 184	2510–3249
Total neutrophils (10^6^/L)	2936 ± 185	2565–3306
Lymphocytes (10^6^/L)	10,473 ± 500	9471–11,474
Monocytes (10^6^/L)	225 ± 30	154–296

SEM = standard error of mean; MCV = mean corpuscular volume; MCH = mean corpuscular hemoglobin; MCHC = mean corpuscular hemoglobin concentration; RDW = red blood cell distribution width.

**Table 2:** Influence of age on the erythrogram of cattle raised in Mozambique, according to statistical characteristics (mean, standard error of mean).

Number of Animals	7	7	11	10	13	8	*p* Value
Age (Months)	0–6	6–12	12–24	24–48	48—72	>72	
**Red blood cells (** **×** **10^12^/L)**	7.54 ± 0.47 ^a^	7.20 ± 0.26 ^a^	7.23 ± 0.48 ^a^	7.53 ± 0.41 ^a^	6.72 ± 0.25 ^a^	6.49 ± 0.25 ^a^	0.2652
**Hemoglobin (g/dL)**	10.45 ± 0.46 ^a^	11.00 ± 0.53 ^a^	10.89 ± 0.38 ^a^	10.99 ± 0.22 ^a^	11.28 ± 0.32 ^a^	11.65 ± 0.19 ^a^	0.3700
**Hematocrit (%)**	27.84 ± 1.26 ^a^	28.80 ± 1.26 ^a^	27.50 ± 0.75 ^a^	29.17 ± 0.73 ^a^	29.10 ± 0.83 ^a^	30.26 ± 1.06 ^a^	0.3712
**MCV (fL)**	37.20 ± 0.94 ^a^	40.34 ± 1.30 ^ab^	39.78 ± 2.10 ^ab^	39.65 ± 1.96 ^ab^	43.63 ± 1.04 ^b^	47.06 ± 2.16 ^c^	0.0048
**MCH (pg)**	14.00 ± 0.50 ^a^	15.30 ± 0.71 ^ab^	15.55 ± 0.70 ^ab^	14.85 ± 0.68 ^b^	16.84 ± 0.40 ^bc^	18.05 ± 0.63 ^c^	0.0006
**MCHC (g/dL)**	37.62 ± 0.95 ^a^	38.01 ± 0.87 ^a^	39.52 ± 0.91 ^a^	37.75 ± 0.52 ^a^	38.79 ± 0.43 ^a^	38.73 ± 1.06 ^a^	0.4673
**RDW (%)**	18.04 ± 0.55	18.28 ± 1.41 ^a^	18.99 ± 1.89 ^a^	16.98 ± 0.48 ^a^	19.43 ± 1.91 ^a^	16.73 ± 0.50 ^a^	0.7294

^a–c^—different letters in the columns mean statistically significant difference between the average value pairs based on Duncan’s test (*p* ≤ 0.05).

**Table 3:** Age influence on leukogram of Landim cattle raised in Mozambique, according to statistical characteristics (mean and standard error of mean).

Number of Animals	7	7	11	10	13	8	*p* Value
Age (Months)	0–6	6–12	12–24	24–48	48–72	>72	
**Leukocytes (10^6^/L)**	12,557 ± 1734 ^a^	18,214 ± 1646 ^b^	16,727 ± 1074 ^bc^	14,740 ± 1116 ^abc^	14,723 ± 971 ^abc^	13,912 ± 1278 ^ac^	0.0232
**Basophils (10^6^/L)**	0 ± 0 ^a^	280 ± 191 ^a^	96 ± 60 ^a^	167 ± 61 ^a^	66 ± 66 ^a^	0 ± 0 ^a^	0.2088
**Eosinophils (10^6^/L)**	359 ± 154 ^a^	1112 ± 374 ^ab^	988 ± 282 ^ab^	891 ± 251 ^ab^	1216 ± 198 ^b^	1588 ± 199 ^b^	0.0470
**Band neutrophils (10^6^/L)**	91 ± 60 ^a^	111 ± 74 ^a^	49 ± 27 ^a^	36 ± 27 ^a^	63 ± 31 ^a^	0 ± 0 ^a^	0.4936
**Segmented neutrophils (10^6^/L)**	2245 ± 318 ^a^	2917 ± 386 ^a^	2733 ± 434 ^a^	2535 ± 277 ^a^	2939 ± 351 ^a^	4008 ± 765 ^a^	0.1700
**Total neutrophils (10^6^/L)**	2336 ± 356 ^a^	3028 ± 351 ^a^	2733 ± 452 ^a^	2571 ± 274 ^a^	3002 ± 352 ^a^	4008 ± 765 ^a^	0.2127
**Lymphocytes (10^6^/L)**	9493 ± 1620 ^ab^	13,252 ± 1781 ^c^	12,473 ± 1055 ^ac^	10,344 ± 918 ^abc^	9389 ± 927 ^ab^	8074 ± 690 ^b^	0.0283
**Monocytes (10^6^/L)**	136 ± 83 ^a^	253 ± 98 ^a^	87 ± 59 ^a^	336 ± 84 ^a^	310 ± 77 ^a^	160 ± 89 ^a^	0.1975

^a–c^—different letters in the columns mean statistically significant differences between the average value pairs based on Duncan’s test (*p* ≤ 0.05).

**Table 4:** Comparison of reference intervals of erythrogram and leukogram of Nguni or Landim cattle, raised in Mozambique, with reference values found in the literature for taurine and Zebuine breeds.

Variable	References Values (95% Confidence Interval) Landim Mozambique	Jersey Brazil [3,4]	Holstein USA [24]	Holstein France [25]	Holstein China [23]	Nelore Brasil [20]	Nelore Brazil [21]
Red blood cells (10^12^/L)	6.78–7.40	5.15–8.09	5.10–7.60	4.80–7.80	4.81–7.54	8.48–9.18	-
Hemoglobin (g/dL)	10.77–11.36	9.16–11.74	8.50–12.20	8.20–13.00	8.60–12.00	11.98–13.00	-
Hematocrit (%)	28.02–29.56	28.10–35.10	22.00–33.00	24.00–39.00	24.50–33.90	36.16–38.78	-
MCV (fL)	39.91–43.02	40.90–57.46	38.00–50.00	41.20–58.70	39.74–57.36	41.41–44.51	-
MCH (pg)	15.27–16.44	13.00–19.74	14.00–18.00	14.30–19.60	14.50–20.10	13.73–14.91	-
MCHC (g/dL)	37.86–39.10	30.57–35.71	36.00–39.00	32.4 0– 35.80	33.80–37.50	32.65–33.95	-
RDW (%)	16.98–19.40	-	15.50–19.70	18.00–26.00	17.90–23.60		-
Leukocytes (10^6^/L)	14,106–16,233	8473–15,221	4900–12,000	4400–10,800	5990–20,470		10,310–16,830
Basophils (10^6^/L)	32–165	0–192	0–300	0–200	50–310		32–60
Eosinophils (10^6^/L)	823–1262	0–750	0–900	100–2200	100–1210		263–979
Band neutrophils (10^6^/L)	25–87	0–202	-	-			-
Segmented neutrophils (10^6^/L)	2510–3249	1146–3768	-	-			-
Total neutrophils (10^6^/L)	2565–3306	1183–3891	1800–6300	700–5100	1650–6800		2310–3600
Lymphocytes (10^6^/L)	9471–11,474	5688–11,744	1600–5600	2000–6200	2240–13,010		6290–13,590
Monocytes (10^6^/L)	154–296	42–306	0–900	100–800	260–1190		10–70

MCV = mean corpuscular volume; MCH = mean corpuscular hemoglobin; MCHC = mean corpuscular hemoglobin concentration; RDW = red blood cell distribution width.

## Data Availability

The original contributions presented in this study are included in the article/Supplementary Materials. Further inquiries can be directed to the corresponding author(s).

## Supplementary Materials

The following supporting information can be downloaded at: https://www.mdpi.com/article/10.3390/vetsci12121124/s1, Figure S1: present the histograms and box plots of Red Blood Cells, Hemoglobin, Hematocrit, Mean Corpuscular Volume (MCV), Mean Corpuscular Hemoglobin (MCH), Mean Corpuscular Hemoglobin Concentration (MCHC), Red blood cell distribution width (RDW), Leukocytes, Basophils, Eosinophils, Band neutrophils, Segmented neutrophils, Total neutrophils, Lymphocytes and Monocytes of Landim Breed.

## Abbreviations

EDTA	Ethylenediaminetetraacetic acid
G	Group
GLM	Generalized linear model
MCHC	Mean corpuscular hemoglobin concentration
MCH	Mean corpuscular hemoglobin
MCV	Mean corpuscular volume
RDW	Red blood cell distribution width
SAS	Statistical analysis system
SEM	Standard error of means
