# Supplementary material for: Reference Values and Effect of Age on Hemogram in Landim Cattle Raised in Extensive System in Districts of Xai-Xai, Limpopo, and Chongoene, Gaza Province, Mozambique
Source: Vet Sci. 2025 Nov 27;12(12):1124. doi: 10.3390/vetsci12121124 (PMC12737348; doi:10.3390/vetsci12121124)

Variable
Red blood cells (x10¹²/ L)

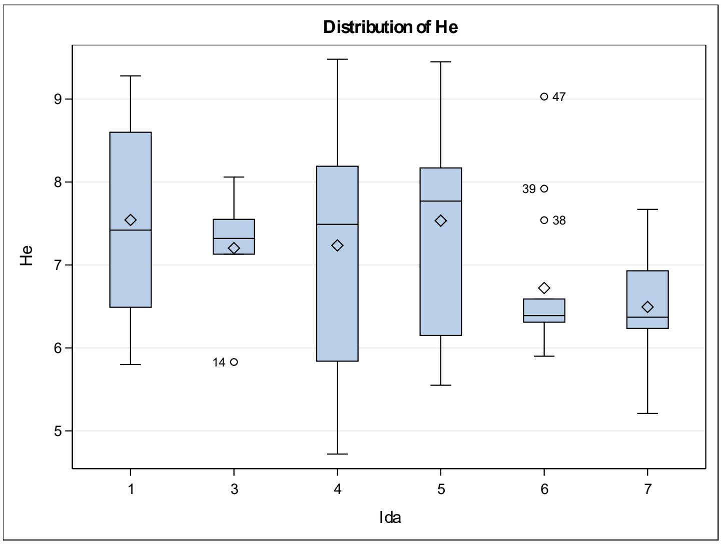

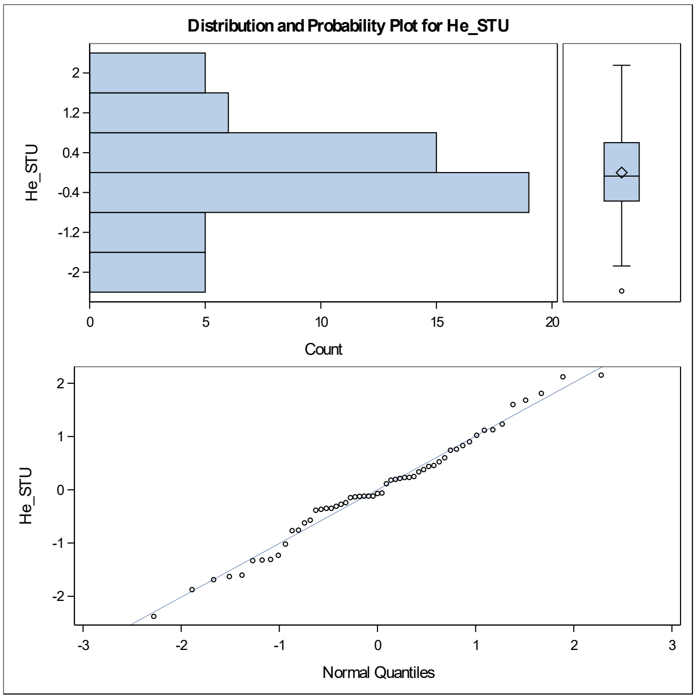

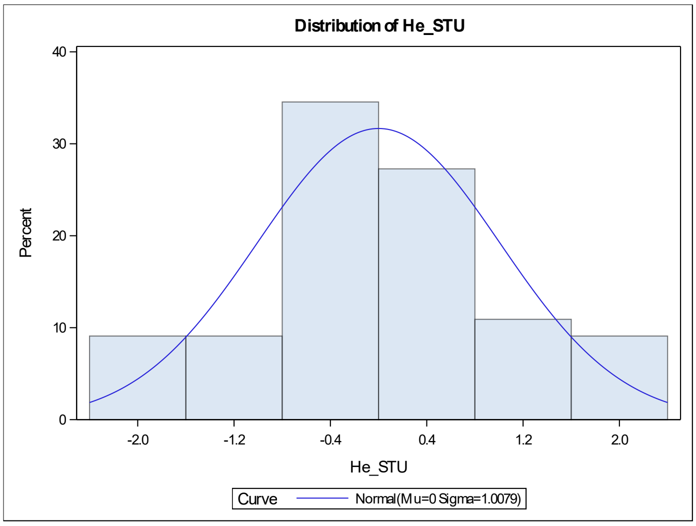

Variable
Hemoglobin (g/dL)

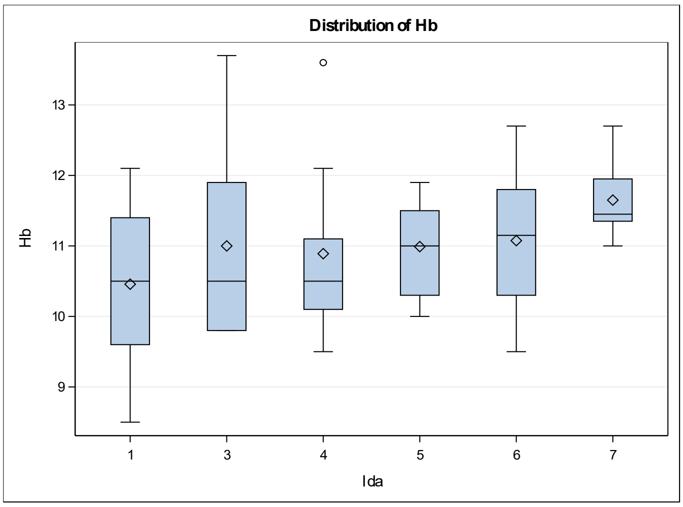

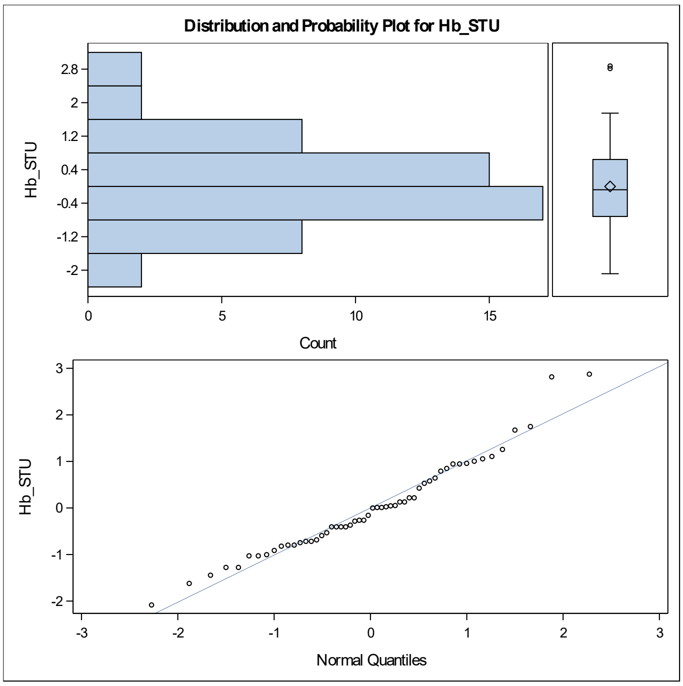

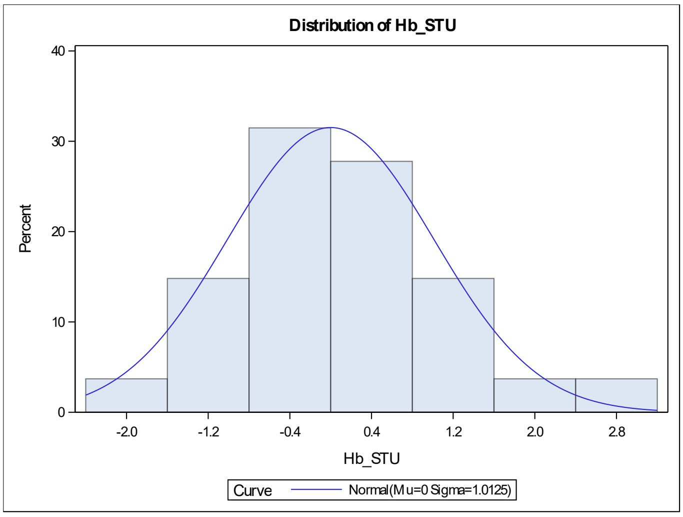

Variable
Hematocrit (%)

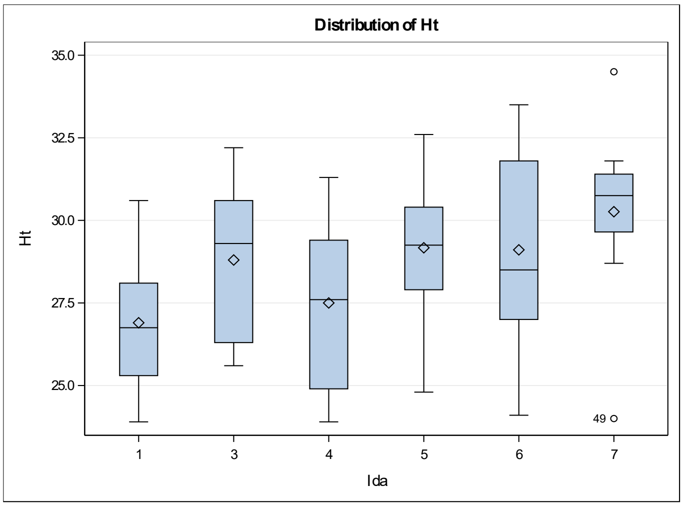

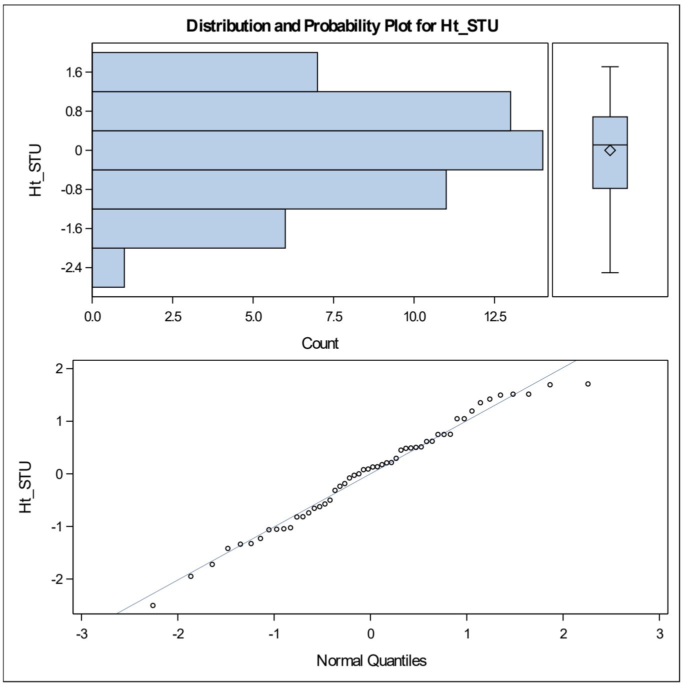

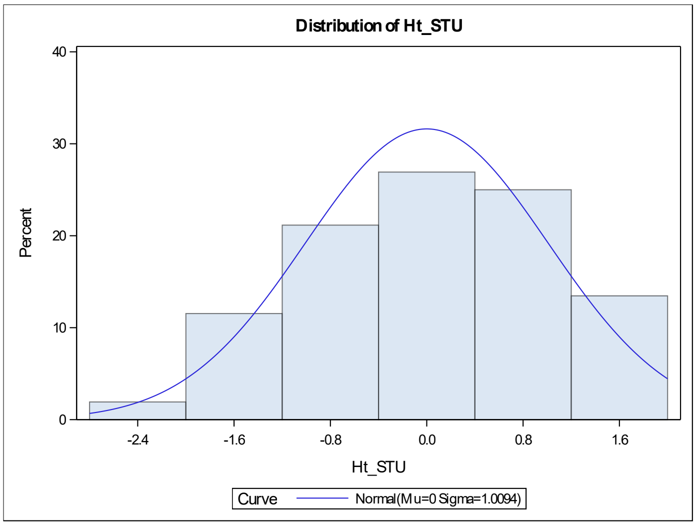

Variable
MCV (fL)

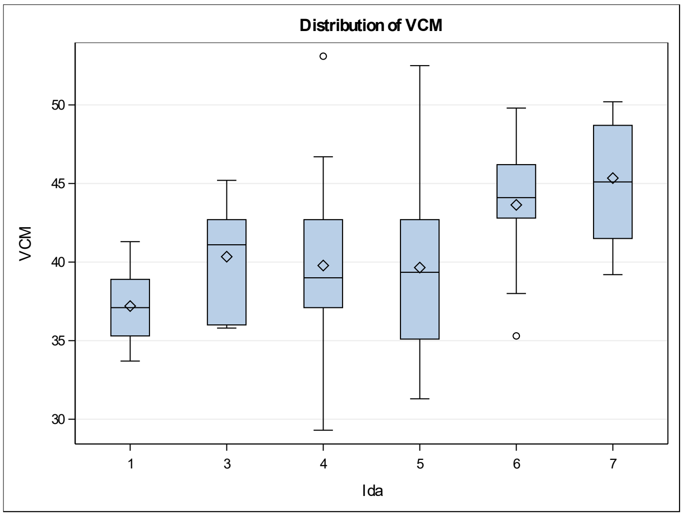

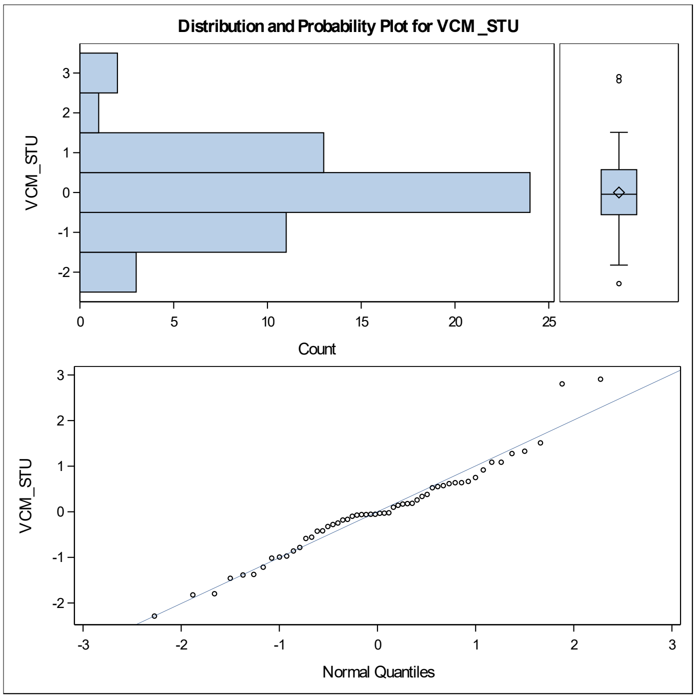

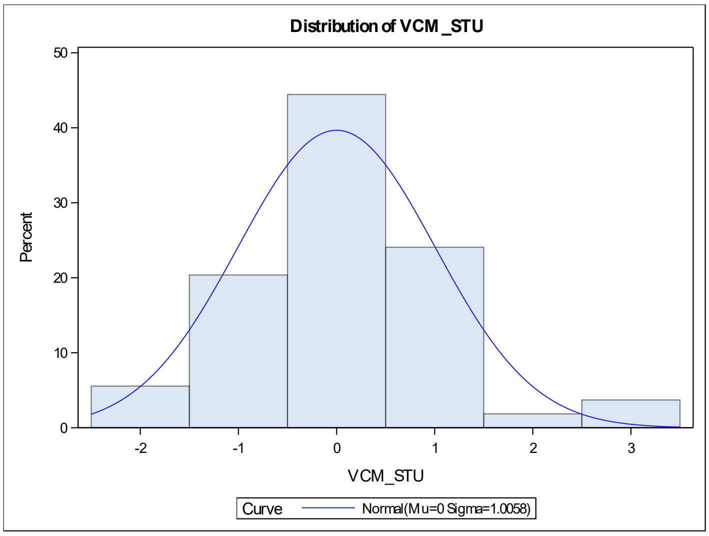

Variable
MCH (pg)

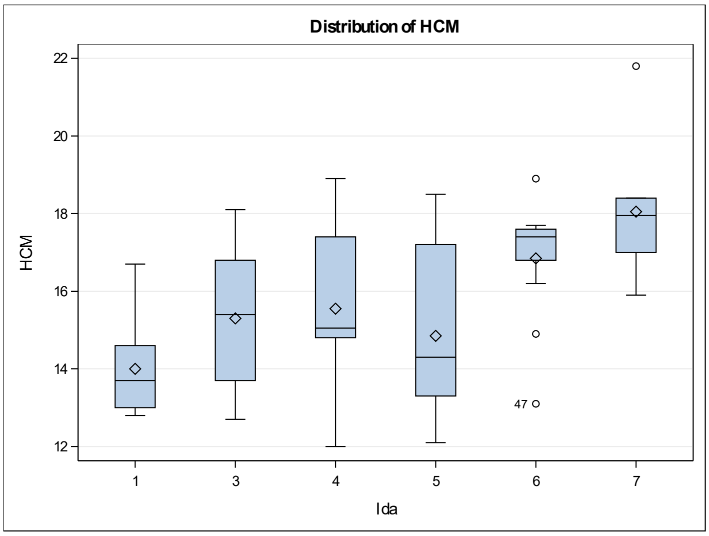

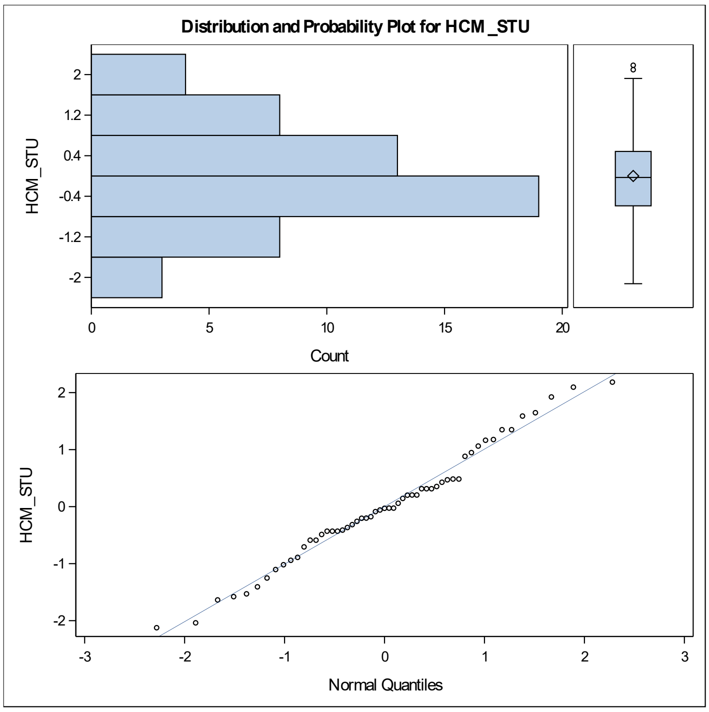

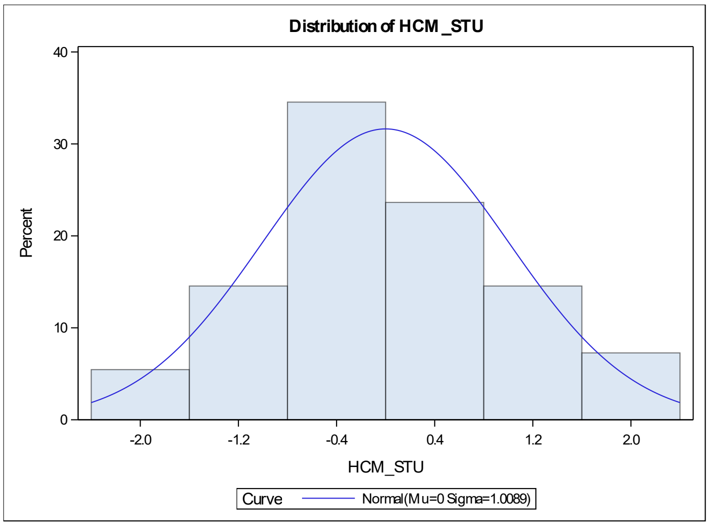

Variable
MCHC **(g/dL)**

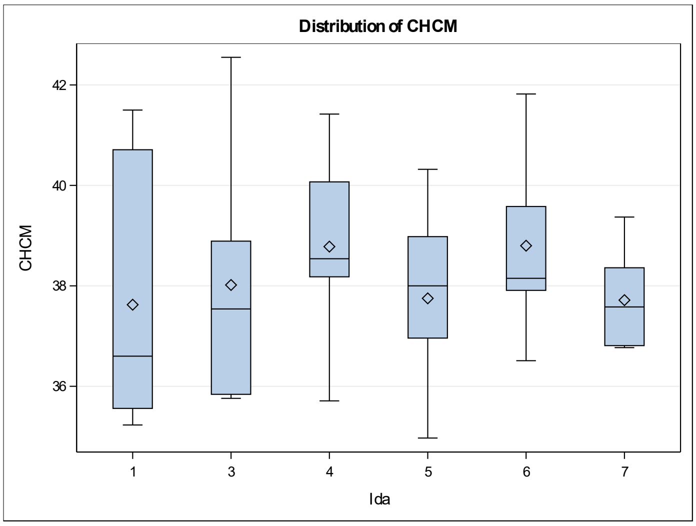

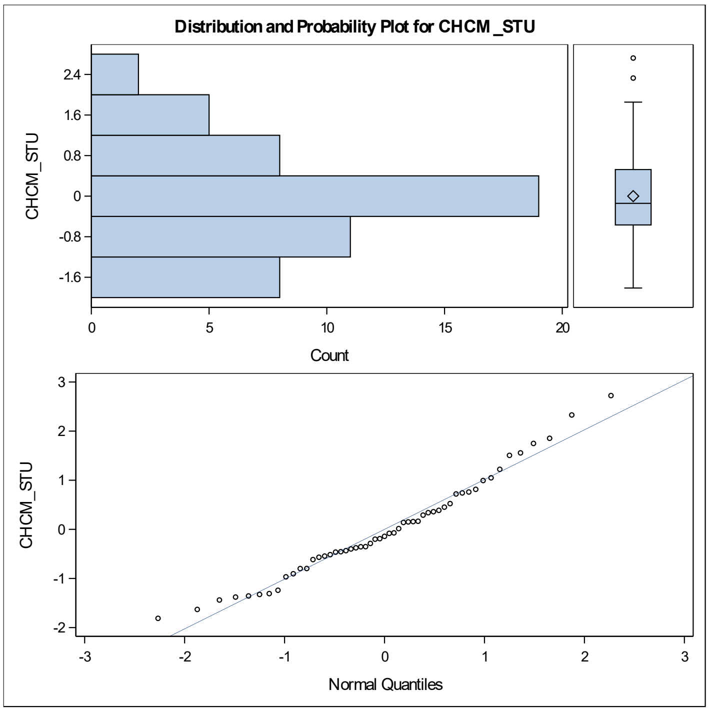

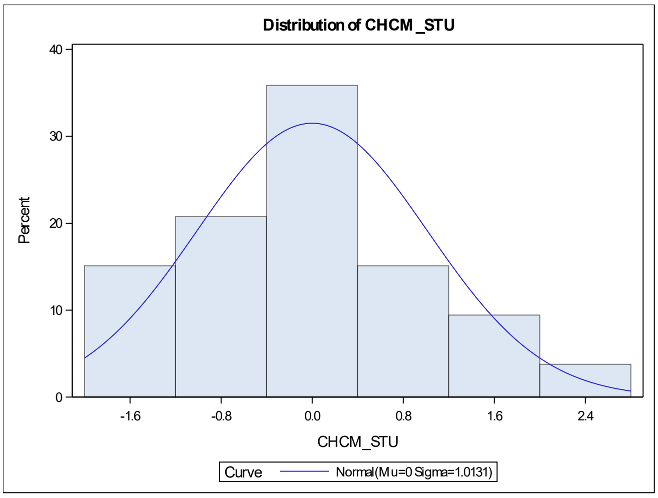

Variable
RDW (%)

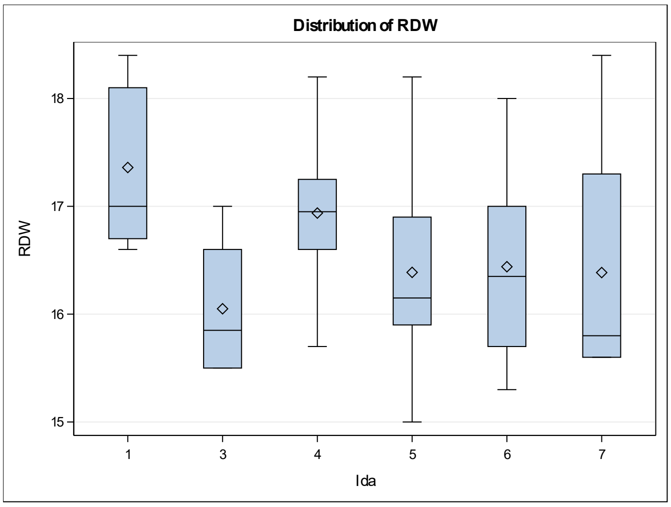

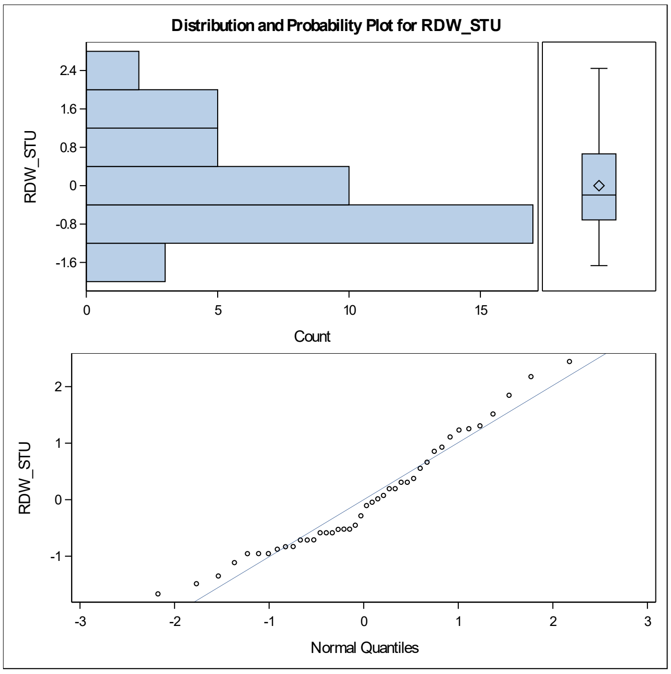

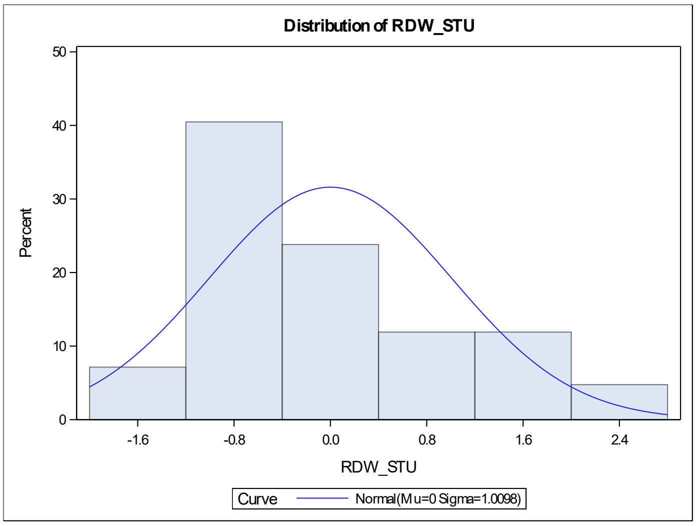

Variable
Leukocytes (10^6/ L)

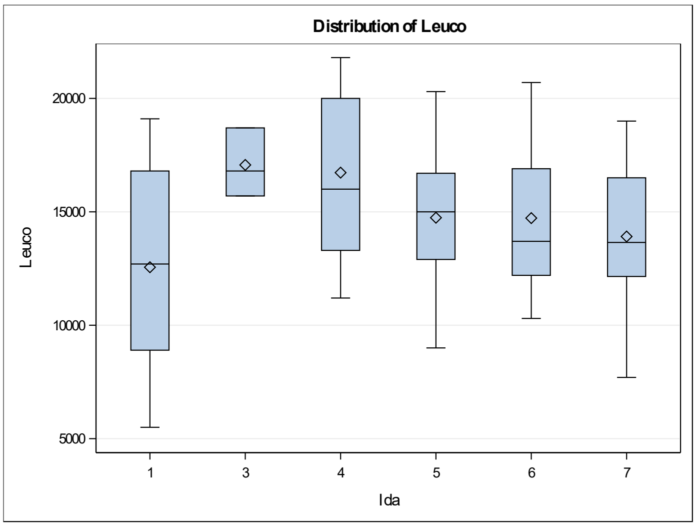

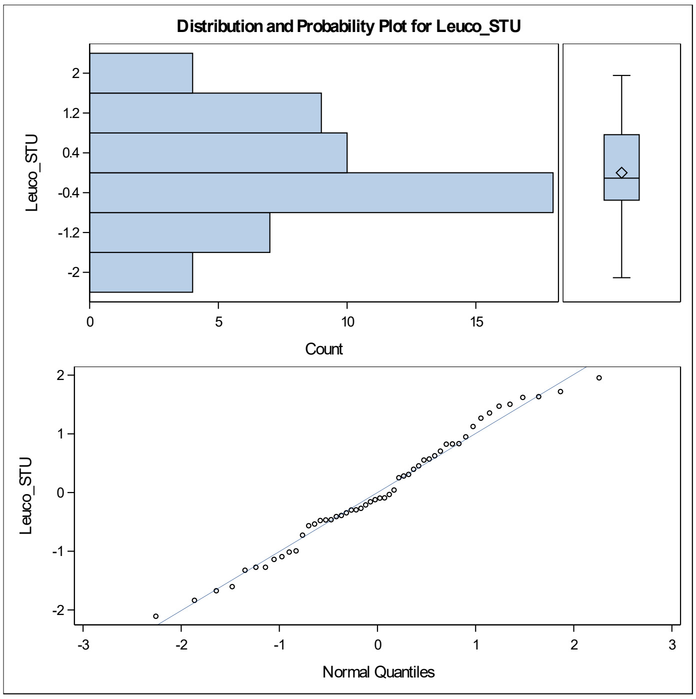

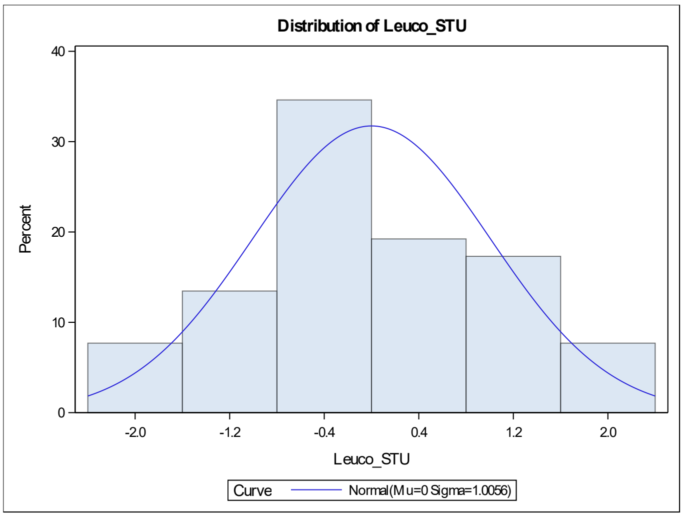

Variable
Basophils (10^6/ L)

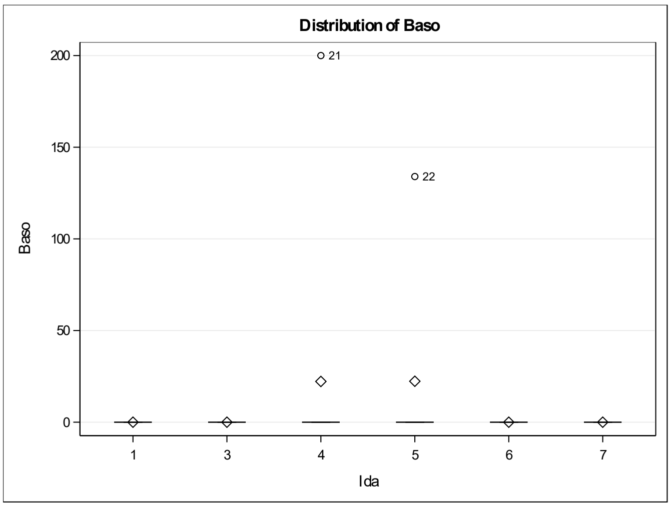

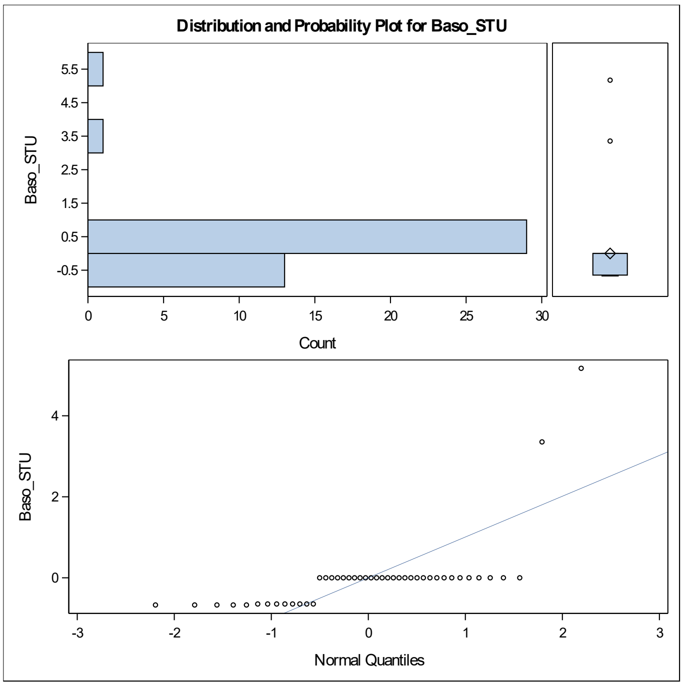

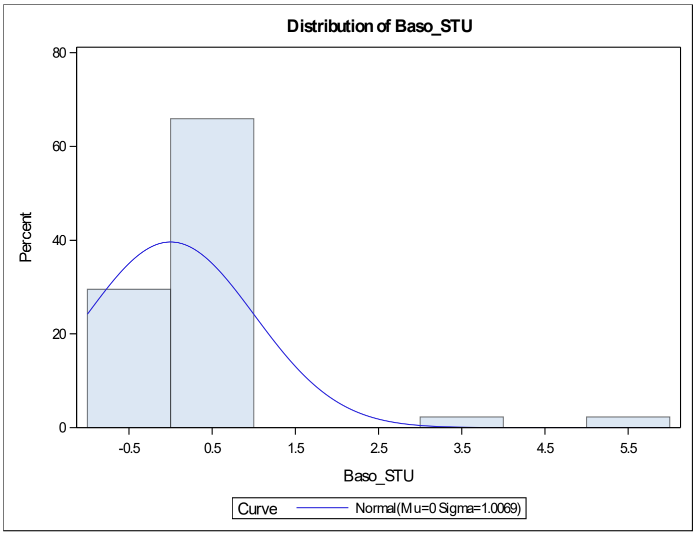

Variable
Eosinophils (10^6/ L)

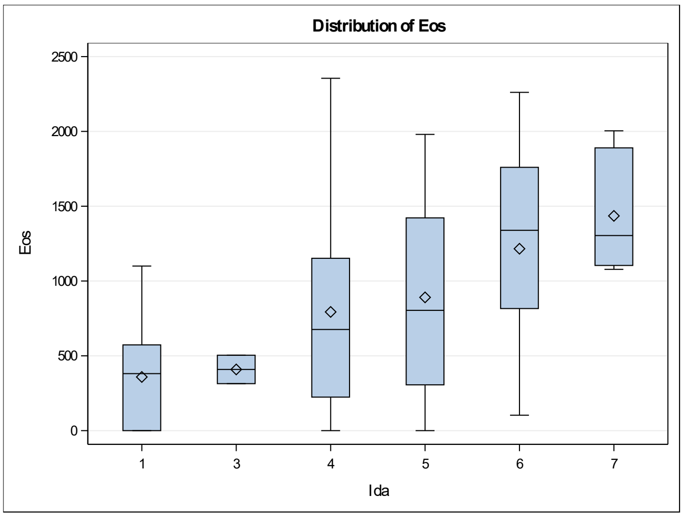

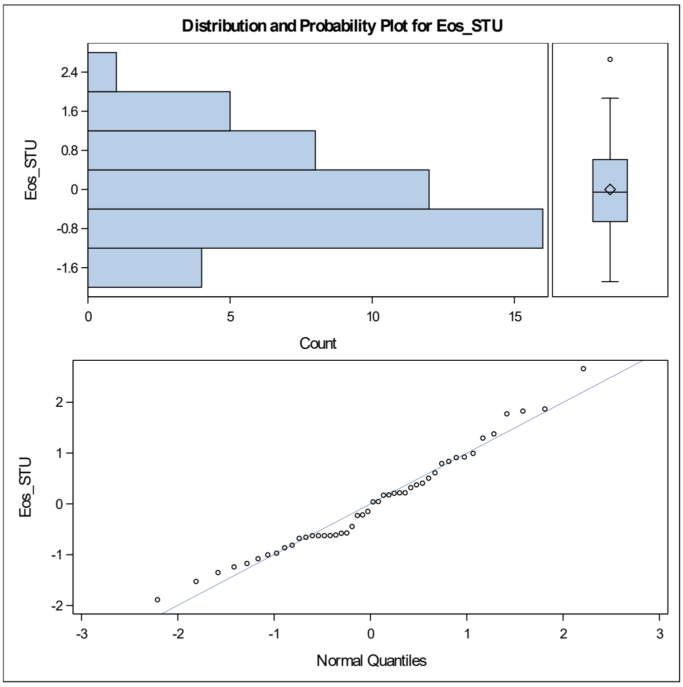

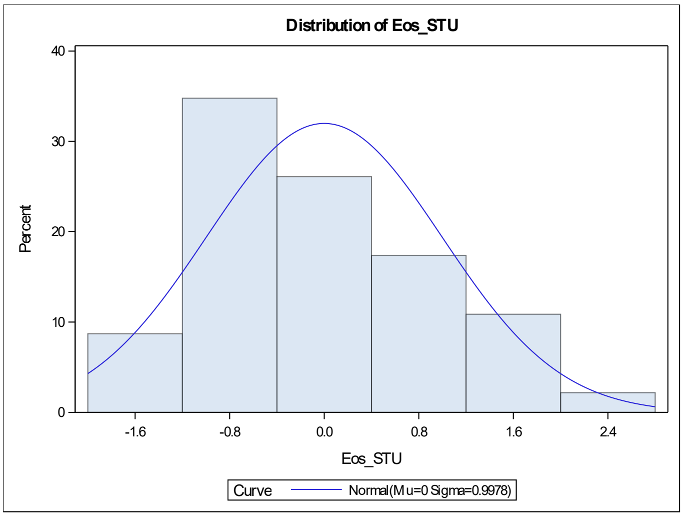

Variable
Band neutrophils (10^6/ L)

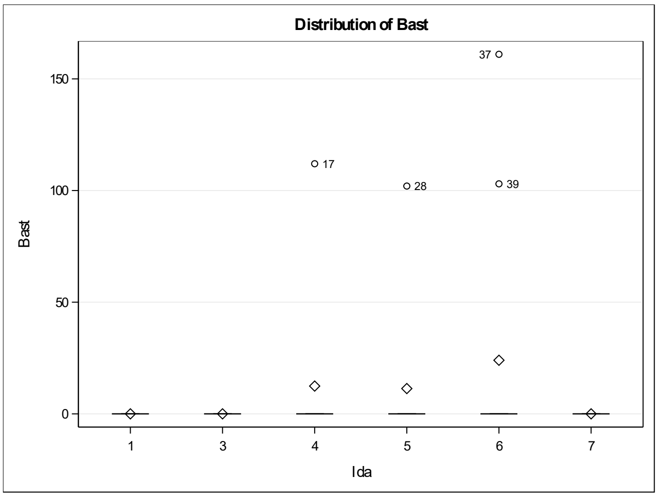

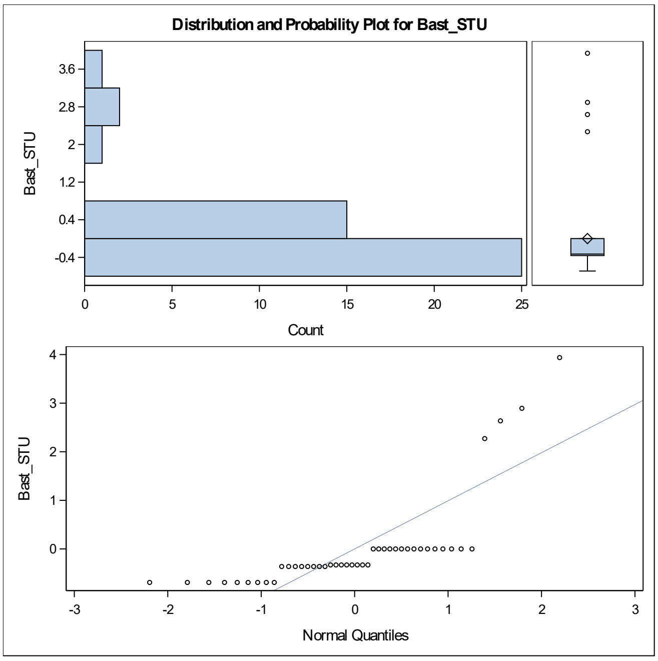

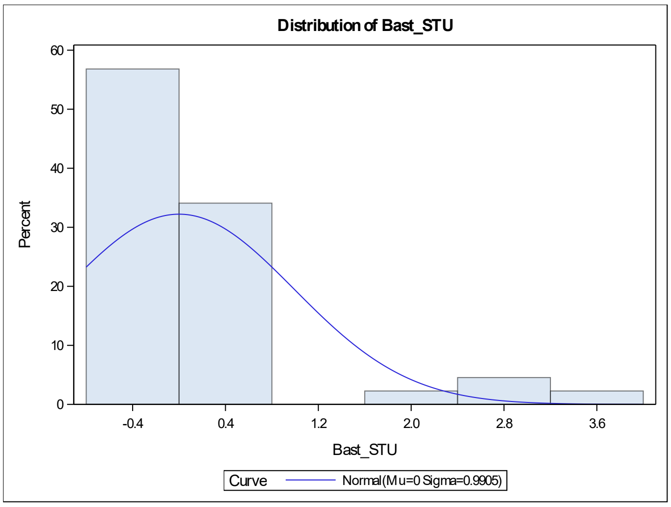

Variable
Segmented neutrophils (10^6/ L)

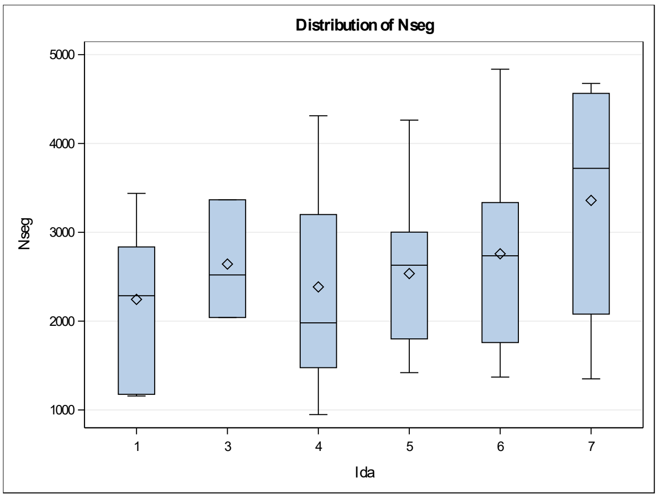

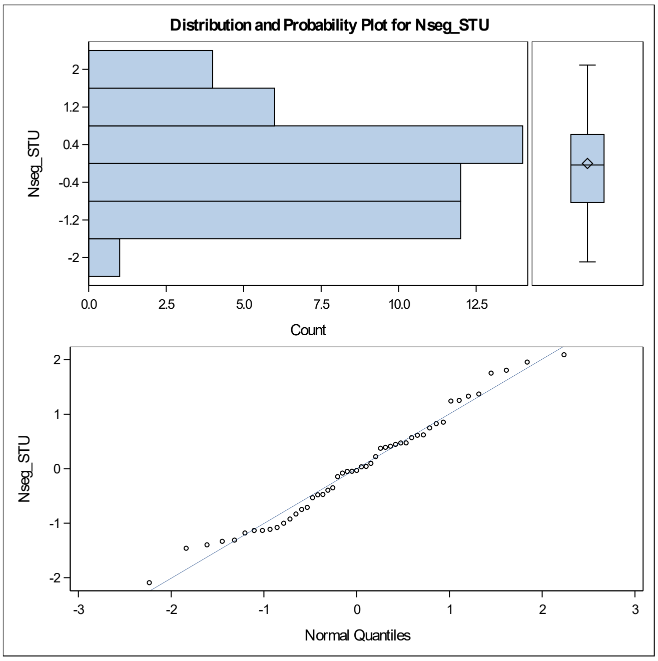

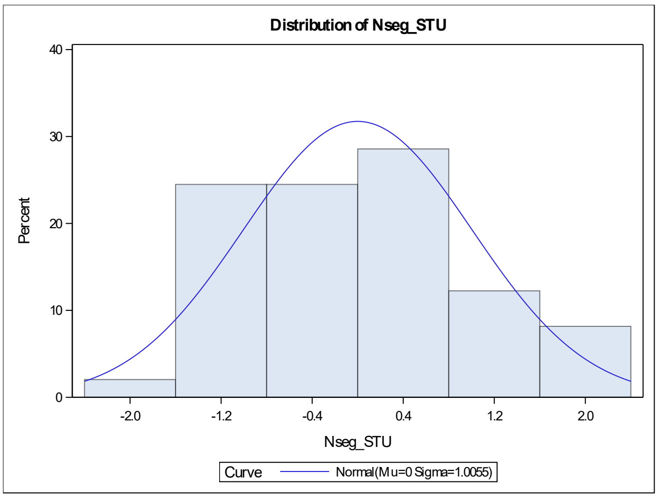

Variable
Total neutrophils (10^6/ L)

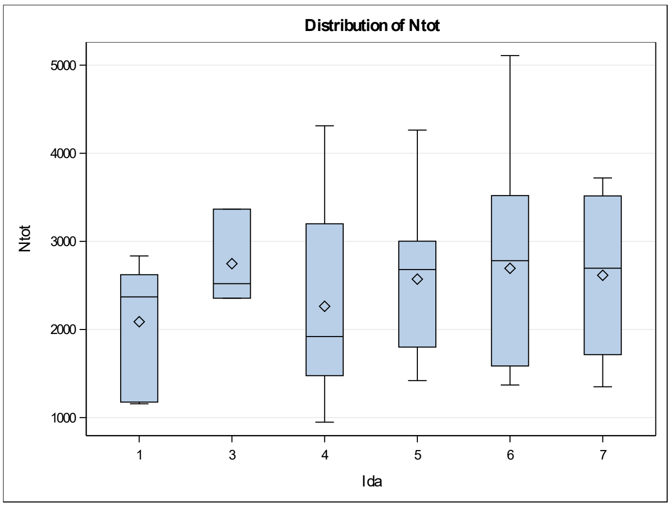

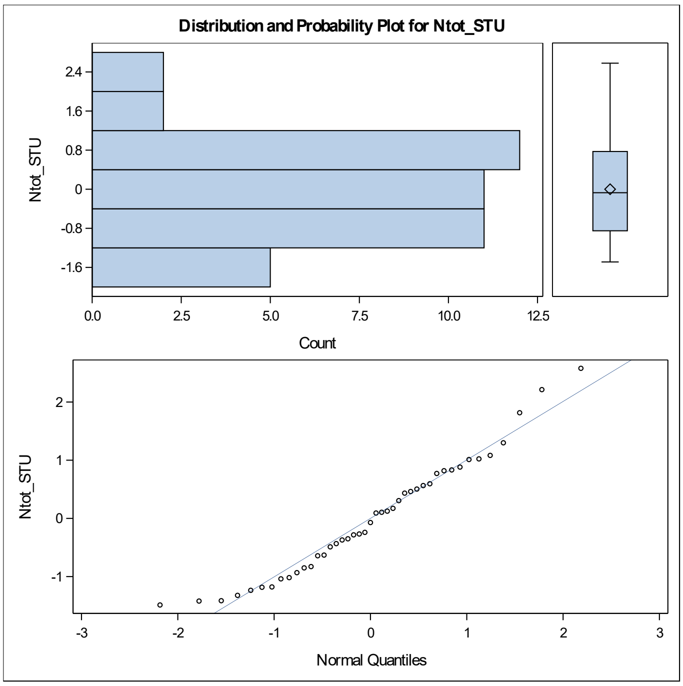

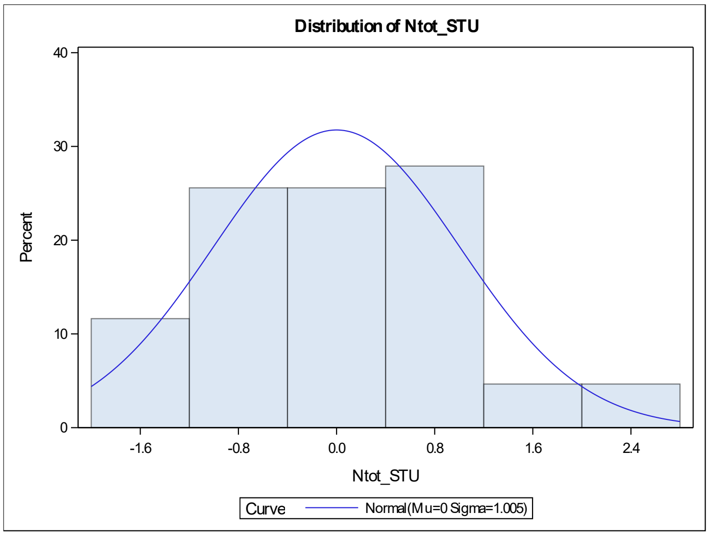

Variable
Lymphocytes (10^6/ L)

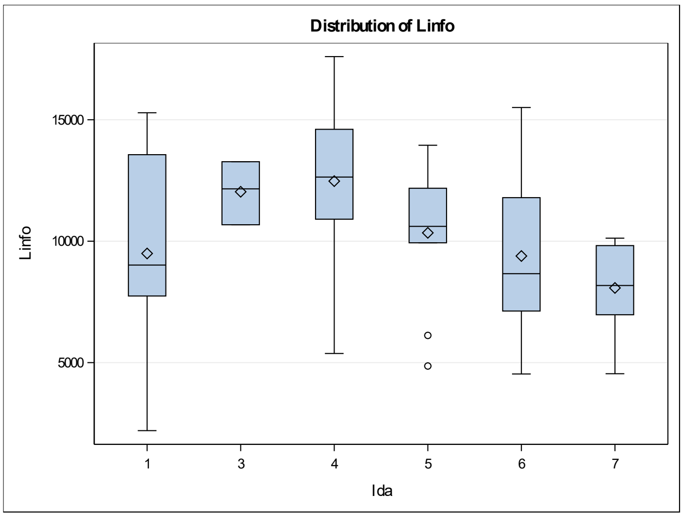

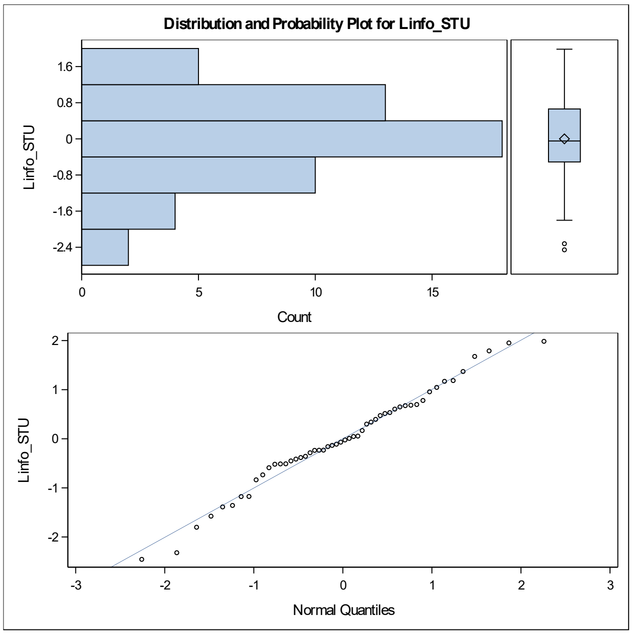

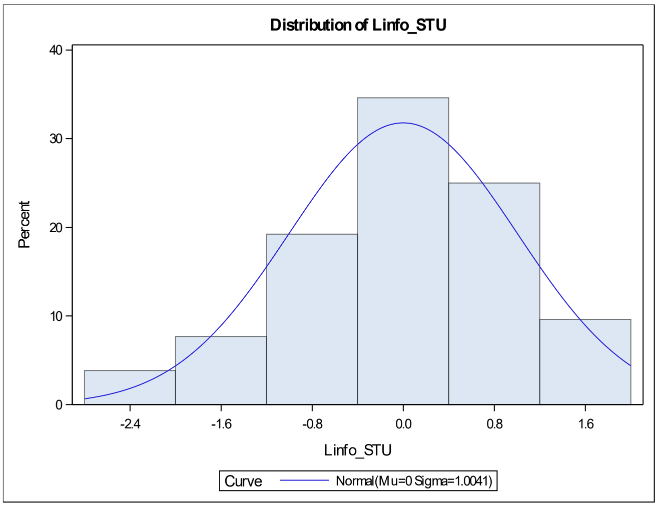

Variable
Monocytes (10^6/ L)

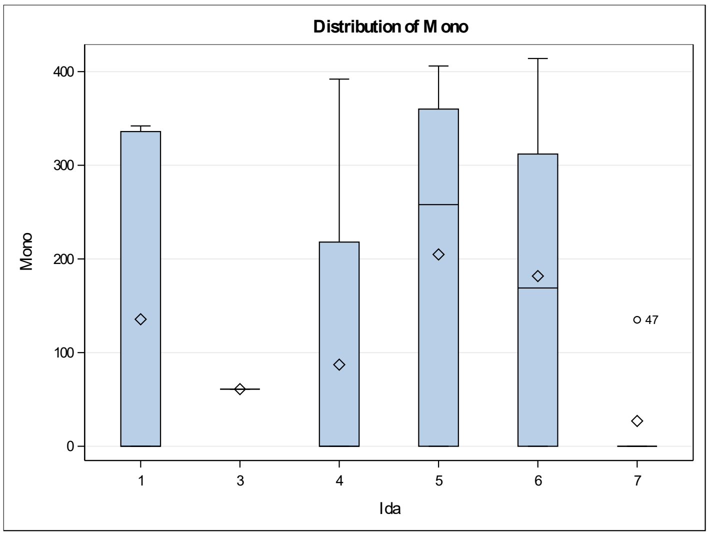

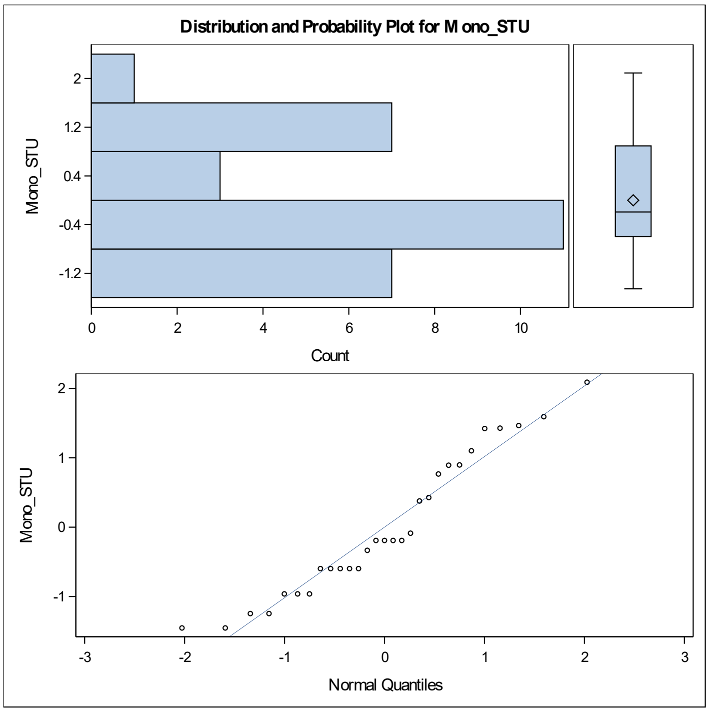

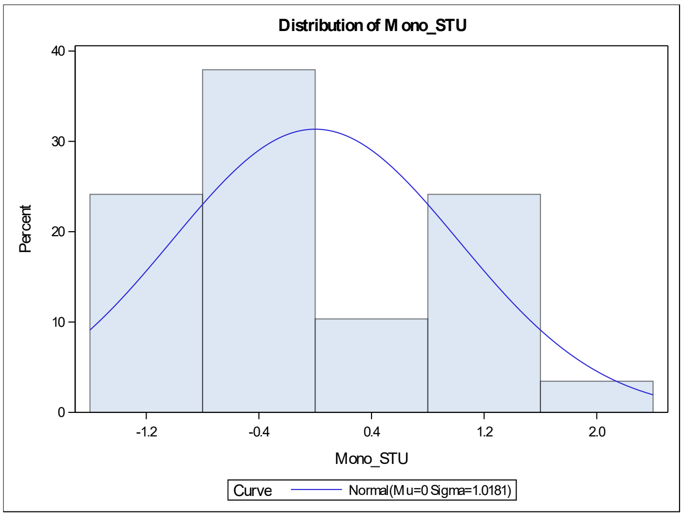

Supplement: Supplementary file 1 [file vetsci-12-01124-s001.zip › vetsci-3893520-supplementary.pdf]
